# Cultured Meat and Malaysia’s Quest for Food Security and Climate Change Mitigation

**DOI:** 10.21315/mjms2023.30.5.19

**Published:** 2023-10-30

**Authors:** Boo Woi Hon

**Affiliations:** Ministry of Health Malaysia, Ampang Baru Community Clinic, Perak, Malaysia

Dear Editor,

I read with interest the recent article by Mahmood et al. (1). The food system reform that the authors advocate, particularly the adoption of a plant-based diet (PBD), to tackle the global food crisis is indeed desirable. However, one novel strategy that also deserves highlighting is the development of cultured meat. Cultured meat (CM), also known as cultivated meat, is a recent innovation whose first prototype appeared in 2013 (2, 3). In essence, CM is clean meat produced in vitro through tissue engineering by harvesting a small amount of live animal tissue and allowing it to proliferate and mature in a growth medium (3). CM negates the need for large-scale animal agriculture, which the authors highlight as not only unsustainable but also a major source of greenhouse gas emissions, which lead to climate change (1).

The level of consumer acceptance of PBD is still far from desirable, further emphasising the role of CM as a transitional product to help reduce traditional meat consumption (4). Additionally, it is possible to customise the nutritional content of CM by modifying the materials used in the medium or by fortifying the CM with various vitamins, trace elements, amino acids and fat. This potentially leads to a healthier meat product and reduces the risk associated with conventional meat consumption, such as cancer, cardiovascular disease and diabetes (2).

As an emerging technology, CM faces numerous barriers, including the high cost of mass production, the difficulty of mimicking the sensory attributes of traditional meat, the challenges of ensuring efficient and safe production, the lack of a regulatory framework, consumer acceptance issues and concerns about the Halal status of the meat (2, 3, 5). Recently, Malaysian Islamic scholars have expressed optimism about CM and have stated that CM can be considered Halal if the cells used for cultivation are derived from Halal slaughtered animals, and the production process does not use animal serum (2, 3).

Despite these challenges, the nascent industry reached a historic milestone when Singapore became the first country in the world to approve the sale of cultured chicken products in 2020 (3, 6). A recent study showed greater acceptance of CM among Singaporeans compared to Americans (7). This is encouraging, as the social fabric of Malaysia is almost identical to that of Singapore. Large-scale, serum-free production is also poised to commence this year in Singapore (6). Hence, the prescribed food system reform mentioned by the authors should ideally anticipate the arrival of CM to our shores by putting in place the necessary legislative/regulatory framework, driving pertinent research, particularly research focusing on the acceptance of CM by the Malaysian population and promoting public awareness of CM.
